# Damping contribution of viscoelastic core on airborne sound insulation performance of finite constrained layer damping panels at low and middle frequencies

**DOI:** 10.1038/s41598-023-42391-9

**Published:** 2023-09-20

**Authors:** Bo Wang, Hequn Min

**Affiliations:** Key Laboratory of Urban and Architectural Heritage Conservation, Ministry of Education, School of Architecture, Southeast University, 2# Sipailou, Nanjing, 210096 China

**Keywords:** Engineering, Aerospace engineering, Civil engineering, Mechanical engineering

## Abstract

The airborne sound insulation performance of finite sandwich panels is often significantly worsened by resonant transmission components in low and middle frequencies. In this paper, damping contribution of viscoelastic core on sound transmission loss (STL) of finite constrained layer damping (CLD) panels is studied in narrow frequency bands. A fully coupled layer-wise approach is used with a generalized high-order shear deformation hypothesis that accounts for all types of deformations in the core. The influence of several parameters is investigated extensively. Results show that the adverse impact of the first-three odd-odd order modes, namely (1,1), (3,1), and (1,3) modes, as well as some higher-order modes on STL cannot be disregarded. The constrained viscoelastic core plays a crucial role in enhancing, or even eliminating, dips of STL spectrum at resonant frequencies. Additionally, it can considerably counterbalance a relatively broadband reduction of STL caused by the inter-modal coupling in middle frequencies. The damping mechanism can be divided into two aspects: (i) the reduction of modal amplitude by vibration energy dissipation, and (ii) the change of bending modal shapes. CLD treatment is a concise and effective way to achieve stable sound insulation performance.

## Introduction

Composite laminates and sandwich structures have the potential to offer superior performance in reducing vibration and noise compared to single-layer components, owing to high specific stiffness and strength^1–3^. In past decades, there has been a growing interest in developing effective models for the design and prediction of lightweight and compact structures that exhibit superior performance^4–6^. Constrained layer damping (CLD), which involves stiffer metal surface layers enveloping a viscoelastic material (VEM) core, is a widely adopted and straightforward treatment for achieving lightweight and high damping compared to acoustic metamaterials^7–10^. Initially proposed by Kerwin et al.^11,12^, it has been the primary method for vibration attenuation in various industries, including aerospace engineering, ocean, and automotive^13–15^. Recently, the application of CLD has expanded with the growing trend of integrating this concept with various composite materials such as fiber-reinforced polymers, to form hybrid metal fiber structures^16–18^. Considerable emphasis is placed on investigating the vibroacoustic response of CLD panels or plate-cavity coupling systems^19,20^, specifically the structure-borne noise radiation behavior under the excitation of harmonic point or line force^21–23^. It is worth noting that almost all existing studies take various types of forces as sources, and the study of acoustic response is regarded as an extended research on vibration control aspects. However, there is limited research on airborne sound insulation performance of CLD structures under various sound field excitation, which is essential to evaluate the suitability of CLD structures as soundproofing panels, and particularly important in aerospace engineering, built environment, and so on. The physical quantity of sound transmission loss (STL) is defined for evaluating the airborne sound insulation capability in audible frequencies.

The work of Kurtze and Watters^24^ marked a groundbreaking contribution to the STL prediction and design of sandwich structures. They utilized the non-dispersive nature of transverse shear waves and proposed a low-shear stiffness core with relatively stiff and thin surfaces, resulting in a high static stiffness and low-weight wall type. However, their approach did not take into account the resonant transmission components caused by dynamic bending stiffness, which is a key limitation. Subsequently, in low and middle frequencies, adverse resonance effects associated with core thickness deformation, resulting in significant dips below the mass law, are identified. Early studies accounted for resonance transmission only by considering the core’s shear and dilatational deformations. (e.g., Dym and Lang^25^, Moore and Lyon^26^). Similarly, Wang et al.^27^ introduced a higher-order approach to estimate the STL of infinite plates, using the assumption of linear variations in the core’s transverse displacement along the coordinates. For predicting the STL of the asymmetric sandwich panel with anisotropic surfaces, a wave impedance model was developed by Zhou et al.^28^. The STL of finite CLD panels with a frequency-independent VEM core was studied by Du et al.^29^, and compared with experimental results. These studies only considered the primary damping mechanism arising from the core’s shear deformation. For thick composite structures or those with large interlayer stiffness differences, such as CLD panels, it has been shown that all types of deformations, including transverse shear, transverse normal, and in-plane deformation, as well as the coupling among them, are indispensable for the accurate analysis of structural dynamic response^30–32^. It is widely recognized that vibroacoustic behavior is closely related to structural dynamic characteristics. Classical lamination theory cannot predict the VEM core response accurately. First-order shear deformation theory (FSDT) may produce inaccurate results when considering the local behavior of thick composite laminates and neglects transverse normal strain ($\varepsilon_{z}$). Therefore, it is of great significance to adopt the refined shell or plate theories [e.g., various the third- or high-order shear deformation theories (HSDTs)]^33,34^. Additionally, it should be emphasized that accurate modal loss factor requires considering the frequency-dependence of VEM core, as pointed out by Carrera et al.^35,36^.

Numerical simulation with finite element (FE) modeling has demonstrated its remarkable efficiency in tackling dynamic problems of complex structures^37,38^. It offers substantial cost savings in the design phase as compared to laboratory testing. When modeling composite structures with the FE mothed, two primary approaches are typically employed: homogenization and discretization. The homogenization approach, also known as the equivalent single-layer approach, involves homogenizing all layers as a single-layer with equivalent dynamic properties, making it an approximate engineering method. On the other hand, the discretization approach allows for local dynamic behavior between layers, making it more accurate. In the traditional discretization approach, the thinner surface layers are modeled with shell elements, and the thicker or softer cores are modeled with brick elements. The two types of elements are coordinated based on their degree of freedoms (DoFs), resulting in a three-dimensional spatial modeling method. The computation execution time significantly rises with the count of DoFs, and the geometric modeling needs to be repeated with every change in layers and thickness. Alternatively, the layer-wise (LW) approach is particularly advantageous since it allows for accurate two-dimensional modeling by describing the layered behavior^39,40^. The axiomatic continuity of displacement fields and transverse stresses are ensured in each successive layer, making the unknown numbers remain constant even with a growing of layers. Thus, this method has become a popular choice for analyzing free vibration of composite CLD structures. Ye et al.^41^ utilized a LW first-order zigzag theory to investigate the STL characteristics of a soft-core sandwich panel, and found that the parameter effect of material properties can significantly affect the occurrence frequencies of STL dips. Moreira et al.^42^ compared the response of sandwich panels obtained using a LW approach with the results achieved using the typical three-dimensional spatial modeling approach, showing that the LW model can correctly capture the VEM core’s high shear deformation patterns. The discussion on the damping of the frequency-dependent VEM interlayer in fiber metal laminates was conducted by Jackstadt et.al^43^ through a LW model with a generalized unified formulation. They found that higher modal damping coefficient makes smaller displacement amplitude and phase shifts in frequency response analysis. D’Ottavio et.al^31^ studied the dynamic behavior of the multiple-core laminates based upon the sub-laminate LW approach within a variable kinematics framework. They demonstrated that the inclusion of transverse normal deformations in the analysis can improve structural damping, particularly for high-order modes. Ferreira et al.^44^ utilized the LW approach of Carrera Unified Formulation^45^ to investigate the free vibration of CLD panels subjected to various commonly used boundary conditions. Other studies were also conducted to examine the STL characteristics of CLD structures. Loredo et al.^46^ derived the Rayleigh–Ritz solution for the LW model of locally damping patched CLD panels. To reduce the number of unknowns, the VEM core was only considered on the assumption of the FSDT, and the fluid loading effect was not included. Results show that mean square velocity decreases correspondingly by adding different VEM cores, and the dips of STL spectrum rise obviously. Assaf et al.^47^ developed a LW model with a three-node triangular plate element for the free vibration and sound insulation performance investigations of CLD panels immersed in light or heavy fluids. A minor limitation of this study is the lack of consideration of the VEM’s frequency-dependence and did not investigation of narrow-band STL. Mejdi et al.^48^ presented a wave spectral FE model for predicting the STL and damping of sandwich panels with various types of VEM cores, with a focus on the effect of compressive deformation on the core. Reddy et al.^49^ analyzed the inherent material damping effect on STL characteristics at resonance frequencies and concluded that damping is important in predicting accurate sound radiating properties, as well as reducing the radiated sound.

Given the literature review above, the primary research focus has been on the free vibration response and the STL prediction of CLD structures. However, a noticeable gap exists in specifically addressing the contribution and underlying mechanism of VEM core to the capability of CLD structures' airborne sound insulation. This is the primary purpose of this work. Upon reviewing previous studies, it is recommended that for higher accuracy in numerical evaluation on the STL of CLD structures, a LW approach that considers sound-structure interaction should be utilized^36,43^, particularly in applications such as aircraft fuselage sidewalls where thin and lightweight soundproofing is required. Therefore, the LW approach based on a generalized high-order kinematic hypothesis is employed to numerically investigate the STL of finite CLD panels in narrow frequency bands.

## Problem description

**Figure 1:**
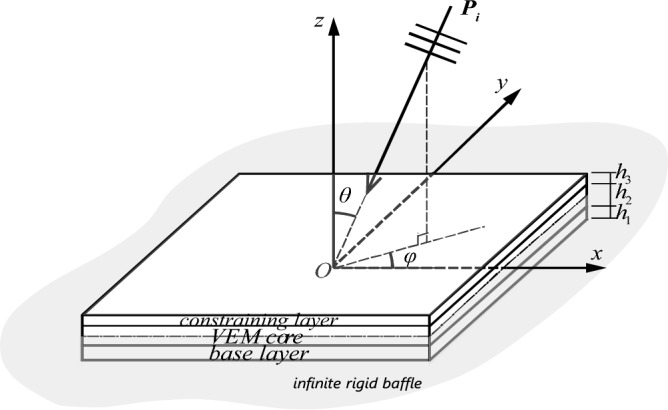
Diagram of the CLD panel under the excitation of plane sound waves, divided by an infinite rigid baffle ($z = 0$) in Cartesian coordinates. The elevation angle is denoted as $\theta \in [0, \, \pi /2]$, and the azimuth angle is denoted as $\varphi \in [0,{ 2}\pi ]$.

**Figure 2:**
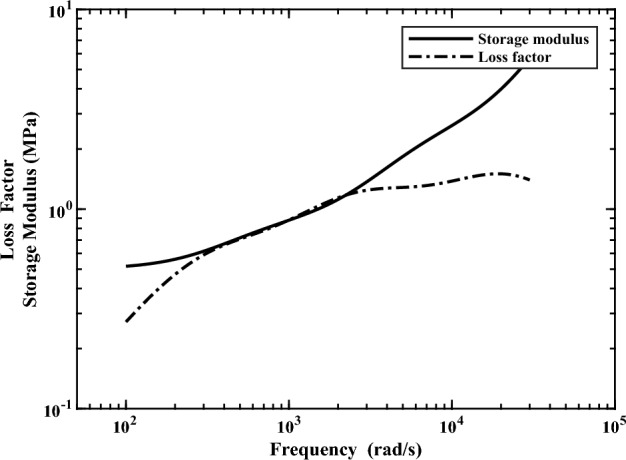
Master curve of VEM typed 3 M-ISD 112 fitted with the ADF model at 27℃.

Figure 1 presents the CLD panel with the in-plane dimension of 0.455 m × 0.376 m under the excitation of plane acoustic waves with varying the sound elevation angle ($\theta$) and azimuth angle ($\phi$). This structure is divided by an infinite rigid baffle ($z = 0$) in Cartesian coordinates with the incident sound field towards the surface of constraining layer. The time harmonic motion ($e^{i\omega t}$) is assumed. The panel comprises two steel face sheets (Young’s modulus $E = 210{\text{ GPa}}$, mass density $\rho = 7800{\text{ kg/m}}^{{3}}$, Poisson’s ratio $\nu = 0.3$, and damping factor $\eta = 0.1\%$) and a VEM core typed 3 M-ISD 112 (mass density $\rho_{c} = 1600{\text{ kg/m}}^{{3}}$, Poisson’s ratio $\nu_{c} = 0.49$). The VEM’s frequency-dependence loss factor and shear modulus can be fitted by the Anelastic displacement fields (ADF) model at 27 ℃^50^ (Fig. 2). Three finite configurations with the same total weight and different thickness combinations are considered: the single-layer panel ($H = 3.0004{\text{ mm}}$), the symmetrical CLD panel ($h_{1} = h_{3} = 1.5{\text{ mm}}$, $h_{2} = 2{\text{ mm}}$), and the asymmetric CLD panel ($h_{3} = 0.25{\text{ mm}}$, $h_{2} = 2 \, {\text{mm}}$, $h_{1} = 2.75{\text{ mm}}$). This employed LW approach accounts for all types of deformations in the VEM core. Furthermore, this study takes into account the full coupling of sound-structure interaction and applies to both light and heavy fluids, despite the air medium parameters of density $\rho = 1.16{\text{ kg/m}}^{{3}}$ and speed $c = 343{\text{ m/s}}$ chosen to investigate. From the objective of this paper described above, the problem to be studied can be simplified to analyze the constrained viscoelastic core’s damping contribution to STL characteristics for CLD panels with the aforementioned parameters, where only sandwich structures with homogeneous layers are investigated. And the corresponding discussions can be reasonably extended to more complex CLD laminates, such as hybrid metal fiber structures, and so on^51,52^. The target frequency of interest in this study falls within the low and middle frequencies (< 2000 Hz), situated under the stiffness-controlled and mass-controlled regions. Within this range, the modal resonance sound transmission path significantly affects the sound insulation. For lightweight metal elastic panels studied in this paper, the coincidence effect predominantly emerges at higher frequencies, while modal resonance has a more pronounced impact in the low and middle frequencies. In the case of the symmetric CLD panel, the critical frequency is approximately 8650 Hz, while for the asymmetric CLD panel, it is around 5040 Hz. Both are well above the target frequencies of interest. Consequently, the influence of coincidence effects on sound insulation remains a topic for future investigation.

## Methods

In this work, the system governing equations of sound-structure interaction can be discretized in frequency domain as^53^

1 $$\left[ {\begin{array}{*{20}c} {{\mathbf{K}}_{S}^{*} - \omega^{2} {\mathbf{M}}_{S} } & { - {\mathbf{C}}_{SA}^{T} } \\ { - \rho_{0} \omega^{2} {\mathbf{C}}_{SA} } & {{\mathbf{K}}_{A} - \omega^{2} {\mathbf{M}}_{A} } \\ \end{array} } \right]\left\{ {\begin{array}{*{20}c} {\mathbf{u}} \\ {\mathbf{p}} \\ \end{array} } \right\} = \left\{ {\begin{array}{*{20}c} 0 \\ {{\mathbf{F}}_{A} } \\ \end{array} } \right\}$$

where ${\mathbf{M}}_{S}$ and ${\mathbf{K}}_{S}^{*} = {\mathbf{K}}_{R}^{{}} + i{\mathbf{K}}_{I}^{{}}$ are structural mass and complex stiffness matrix, and ${\mathbf{K}}_{I} = {\mathbf{K}}_{I} (\omega )$ is also frequency-dependence. ${\mathbf{C}}_{SA}$,${\mathbf{M}}_{A}$, and ${\mathbf{K}}_{A}$ are the acoustic coupling matrix, mass matrix, and stiffness matrix. ${\mathbf{F}}_{A} { = }2\overline{p}_{i} \int_{S} {{\mathbf{p}}_{i} (\omega ){\mathbf{N}}^{{\text{T}}} {\text{d}}S}$ is the vector of sound pressure with the amplitude $\overline{p}_{i}$.

On the interface, the continuous condition of the normal velocity of vibrated structural surface and surrounding medium particles can be described as

2 $${\mathbf{n}}^{T} \rho_{0} \omega^{2} {\mathbf{u}} = - {\mathbf{n}}^{T} \nabla {\mathbf{p}}{\text{ on }}\Gamma_{SA}$$

where the unit normal vector ${\mathbf{n}}$ is directed towards the air. The interaction interface between air and CLD panel is $\Gamma_{SA}$.

**Figure 3:**
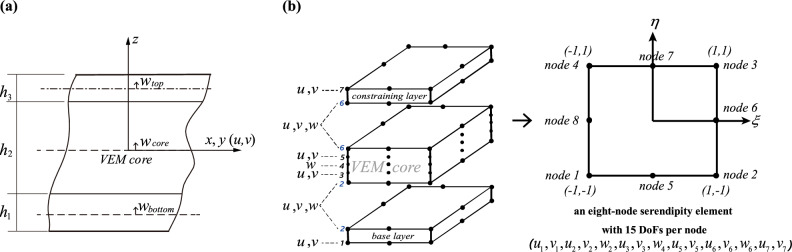
Diagram of the LW model's kinematic relations and the assembly of FE element nodes. (**a**) The motion of displacement fields. (**b**) Local coordinates of the adopted eight-node serendipity element.

The $C_{z}^{0}$-continuous displacement field ${\mathbf{u}} = [u_{x} ,u_{y} ,u_{z} ]^{T}$ with the transverse stress ${{\varvec{\upsigma}}} = [\sigma_{zx} ,\sigma_{zy} ,\sigma_{zz} ]^{T}$ are all considered and satisfied in the LW model. Figure 3 presents the kinematic relations of the structure. The through-thickness displacement field regarding the generalized HSDT can be expressed through Taylor’s series expansion as

3 $$\left\{ {\begin{array}{*{20}c} {\overline{u}_{i} (x,y,z) = u_{i} (x,y) + zu_{i1}^{*} + z^{2} u_{i2}^{*} + \cdots + z^{m} u_{im}^{*} } \\ {\overline{v}_{i} (x,y,z) = v_{i} (x,y) + zv_{i1}^{*} + z^{2} v_{i2}^{*} + \cdots + z^{m} v_{im}^{*} } \\ {\overline{w}_{i} (x,y,z) = w_{i} (x,y) + zw_{i1}^{*} + z^{2} w_{i2}^{*} + \cdots + z^{n} w_{in}^{*} } \\ \end{array} } \right.$$

where the layer number is represented with the subscript $i = 1,2,3$. $u_{i}^{{}}$, $v_{i}^{{}}$, and $w_{i}^{{}}$ are the mid-plane’s longitudinal and transverse displacements for each layer. $w_{i1}^{*}$ is transverse displacement linear term. $v_{i1}^{*}$ and $u_{i1}^{*}$ are the *x*- and *y*-axis rotational angles. The higher-order motion of displacement fields is written in Taylor’s series form: $w_{ik}^{*}$ with $k = 1,...,n$, $v_{ij}^{*}$ and $u_{ij}^{*}$ with $j = 1,...,m$. One can choose different expansion orders flexibly according to the specific problem, to balance the calculation efficiency and accuracy. A mixed form of the presented formula is used for the CLD configurations in this study. For the stiffer metal surface layer ($i = 1,3$), the expansion order of $\overline{u}_{i}$ and $\overline{v}_{i}$ is $j = 1$, and the expansion order of normal displacement $\overline{w}_{i}$ is $k = 0$, which is equivalent to the so-called FSDT. For the VEM core ($i = 2$), to accurately capture its deformation, the expansion order of $\overline{u}_{i}$ and $\overline{v}_{i}$ is $j = 3$, and the expansion order of normal displacement $\overline{w}_{i}$ is $k = 2$. The assumption is discretized by an 8-node quadratic shell element with 15 DoFs per node, and the schematic of nodes arrangement is shown in Fig. 3b, in which the condition of interlayer continuity is formed through points 2 and 6.

The vector of nodal displacements ${\mathbf{U}}_{p}^{e}$ can be assembled by shape functions to form the vector of element displacement and is given by

4 $${\mathbf{U}}^{{\text{e}}} = \sum\limits_{i = 1}^{8} {N_{i} (\xi ,\eta )} \left\{ {\overline{{u_{i} }} } \right\}{ = }{\mathbf{N}}_{U} {\mathbf{U}}_{p}^{e}$$

where the $i{\text{ - th}}$ nodal displacement vector and its associated shape function are $\left\{ {\overline{{u_{i} }} } \right\} = \left[ {u_{1} ,v_{1} ,u_{2} ,v_{2} ,w_{2} ,u_{3} ,v_{3} ,w_{4} ,u_{5} ,v_{5} ,u_{6} ,v_{6} ,w_{6} ,u_{7} ,v_{7} } \right]^{T}$ and $N_{i} (\xi ,\eta )$. Then, the structural dynamic equation is deduced from Hamilton’s principle. To clarify, the detailed derivation process for the elemental mass and stiffness matrices can be found in Supplementary Appendix A.

The dynamic equation can be derived through connecting all elemental mass and stiffness matrices to global coordinate system and is given by

5 $${\mathbf{M}}_{S} {{\ddot{\mathbf u}}} + {\mathbf{K}}_{S} {\mathbf{u}} = {\mathbf{F}}_{A}$$

Since the VEM core’s frequency-dependence is considered, the resulting eigenvalue problem is nonlinear, and solved through the iterative procedure^31^.

The simply supported and clamped are two frequently-used constraint boundary conditions. For the first case, the edges parallelly oriented with the *x*-axis are $w = 0$ and $u = 0$, and the edges parallelly oriented with the *y*-axis are $w = 0$ and $v = 0$. For the latter, the expression is $u = v = w = 0$.

It is feasible to utilize complex modulus to describe VEM core’s frequency-dependence. The shear modulus is expressed as

6 $$G^{*} (\omega ) = G{\prime} (\omega ) + iG^{^{\prime\prime}} (\omega )$$

where the storage and loss modulus are $G{\prime} (\omega )$ and $G^{^{\prime\prime}} (\omega )$. $\eta = G^{^{\prime\prime}} (\omega )/G{\prime} (\omega )$ is the damping loss factor. A three-series form of the ADF model is adopted, which can characterize the viscoelasticity accurately by elastic and anelastic parts, and is very suitable for implementation in the FE solution process. The expression is as follows^50^

7 $$G^{*} (\omega ) = G_{0} \left( {1 + \sum\limits_{j = 1}^{3} {\frac{{\Delta_{j} \omega }}{{\omega - i\Omega_{j} }}} } \right)$$

where $G_{0}$ is the shear modulus in the static regime. The strength and relaxation time are $\Delta_{j}$ and $1/\Omega_{j}$.

In the present methodology, the fluid loading effect is considered for higher accuracy, though they are usually neglected for a light fluid. The radiated sound is calculated through the method of Rayleigh integral, which has the advantage that there are no additional unknowns beyond those arising from the structural FE solution procedure. Thus, the radiated sound pressure is

8 $$\hat{p}_{r} ({\mathbf{r}}) = \rho \omega^{2} \int_{{S_{e} }} {G\left( {{\mathbf{r}},{\mathbf{r}}_{0} } \right)v_{n} \left( {{\mathbf{r}}_{0} } \right){\text{d}}S_{e} } ,\quad z < 0$$

where $G = e^{ - ikR} /2\pi R$ is the free-field Green’s function, and satisfying the rigid baffle boundary condition $\partial G/\partial n$. $R$ is the space dimension from the source position (${\mathbf{r}}_{0}$, ${\mathbf{x}}_{0}$) on structural surface to receiver position (${\mathbf{r}}$, ${\mathbf{x}}$) in sound field.

The STL is expressed as

9 $${\text{STL}} = 10\log \left( {\frac{{W_{i} }}{{W_{r} }}} \right)$$

where the transmitted and incident power is represented as $W_{r}$ and $W_{i}$. The incident power sound can be written as

10 $$W_{i} (\theta ,\varphi ) = \frac{{\left| {p_{i}^{{}} } \right|^{2} S\cos \theta }}{2\rho c}$$

where $\rho c$ and $S$ are the air characteristic impedance and the structural surface area. $p_{i}$ represents the incident sound pressure.

For the plane sound excitation with normal or oblique elevation angles, $p_{i}$ is written as

11 $$p_{i} (x,y,z) = \widehat{P}_{i} \exp \left[ { - {\text{i}}\left( {k_{x} x + k_{y} y + k_{z} z} \right)} \right]$$

For the condition of diffuse sound excitation, it is common to assume that the sound field is the sum of large numbers of uncorrelated plane waves moving in random directions.

In addition, the transmitted sound power can be calculated as

12 $$W_{r} (\theta ,\varphi ) = \frac{1}{2}\int_{{\Gamma_{SA}^{{}} }} {\Re (\hat{p}_{r} v_{n}^{*} )d\Gamma_{SA}^{{}} } = \frac{{S_{e} }}{4}\Re \left\langle {{\mathbf{v}}_{n}^{H} \left( {{\mathbf{Z}}^{H} {\mathbf{Z}}} \right){\mathbf{v}}_{n}^{{}} } \right\rangle = {\mathbf{v}}_{n}^{H} {\mathbf{Rv}}_{n}^{{}}$$

where the complex conjugate and Hermitian transpose are represented as the superscript * and $H$.  $\hat{p}_{r}$ is sound pressure, and  $v_{n}^{{}}$  are the complex normal velocity amplitudes. $S_{e}$  is each element’s area. ${\mathbf{R}}$  is the real part of radiation impedance matrix ${\mathbf{Z}}$, named radiation resistance matrix. Meanwhile, the fluid loading effect can be indicated by the imaginary part of  ${\mathbf{Z}}$. The method to deduce radiation impedance matrix can be found detailly in Refs.^47,54^.

## Model validation

In this section, two cases are conducted to verify the accuracy of the employed numerical model. Firstly, the free vibration analysis is conducted on a sandwich panel with varying the aspect ratios from thin to thick, where mesh convergence is also examined. Secondly, the STL of CLD panel with a frequency-independent VEM core is investigated and compared with published experimental data.

### Case 1: free vibration of sandwich panel varying the aspect ratios

The free vibration analysis is conducted on a square sandwich panel (0°/90°/0°) under the simply supported condition. Each layer has the same thickness and material. The total thickness and width of the panel are represented by *h* and *a*, respectively. The aspect ratio between the width and total thickness of the panel can be written as $a/h$. Here, the aspect ratios ($a/h$) are chosen as 2, 5, and 10, respectively. The non-dimensional parameters are: $E_{1} /E_{2} = {40}$, $E_{3} = E_{2}$, $\nu_{23} = 0.49$, $\nu_{12} = \nu_{13} = 0.25$,$G_{12} /E_{2} = G_{13} /E_{2} = 0.6$, $G_{23} /E_{2} = 0.5$.

To cope with all conditions of the structural thickness varying from thin to thick, the in-plane and through-thickness displacement fields defined by Eq. (3) for the core layer (*i*=2) are chosen as cubic and quadratic interpolations, namely, ($m$, $n$) = (3, 2). And the non-dimensional fundamental frequency ($\varpi_{mn} = \omega_{mn} \sqrt {\rho h^{2} /E_{2} }$) is calculated.

**Figure 4:**
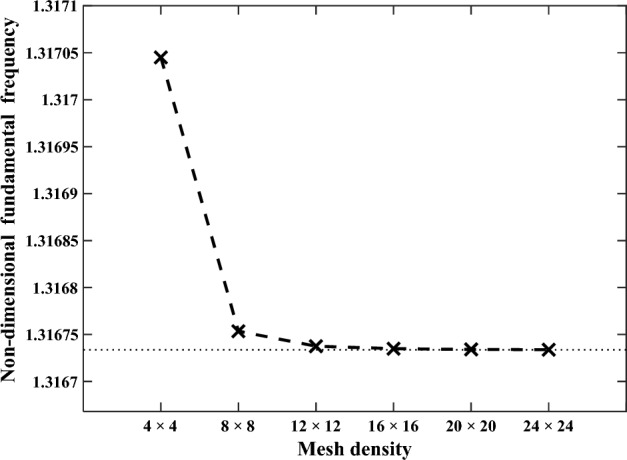
Mesh convergence examination of the sandwich panel with the aspect ratio ($a/h$) of 2.

Firstly, mesh convergence is studied based on the aspect ratio of 2. This selection can ensure a surplus of mesh generation for other small thickness situations. As is shown in Fig. 4, there is a rapid convergence as the mesh density increases. A mesh density of 20 × 20 provides a good balance between accuracy and computational cost and is therefore considered for subsequent analyses.

Table 1 presents the comparison results of $\varpi_{mn}$ with various solutions in the literature. As the increase of aspect ratios, the natural frequency decreases. Thicker panels have a higher natural frequency, and vice versa. The results obtained with FSDT show a significant deviation from the exact solution for thicker laminates^55,56^, indicating that it is not suitable for such cases. In contrast, the calculated data of the present model agree well with those from different HSDT models (Sayyad et al.^57^, Matsunaga et al.^58^), as well as the results of Ferreira et al.^59^, which indicates the accuracy of the present model.

**Table 1:** The non-dimensional fundamental frequency with various aspect ratios.

$a/h$	Method					
	Present (3, 2)	Exact (Reddy^55^)	HSDT (Sayyad et al.^57^)	HSDT (Matsunaga et al.^58^)	Modified-FSDT (Ferreira et al.^59^)	FSDT (Xiang et al.^56^)
2	1.31673	1.30125	1.35346	1.2981	1.30275	1.38325
5	0.40845	0.41160	0.41176	0.4101	0.41228	0.42792
10	0.14721	0.14766	0.14720	0.1471	0.14804	0.14753

### Case 2: STL of a CLD panel with frequency-independent VEM core

**Figure 5:**
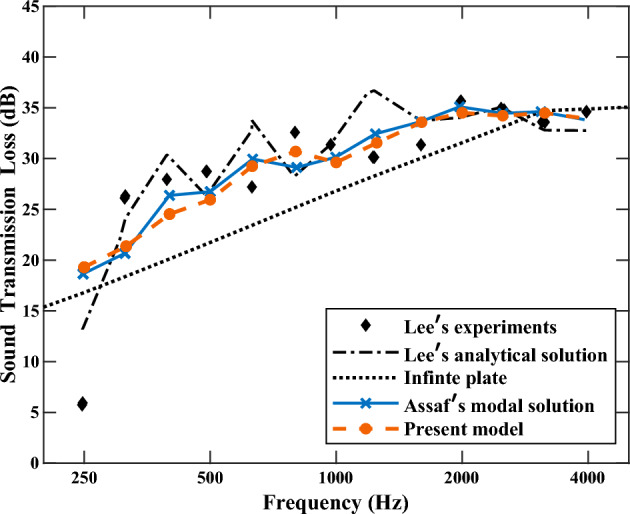
STL spectra of the present model, the experiments and analytical solution of Lee et al.^60^, and the direct modal solution of Assaf et al.^47^.

The prediction ability of the present LW model for STL is investigated by considering a CLD sandwich panel. This specific configuration has been previously studied analytically and experimentally by Lee and Kondo^60^, and then through the modal summation approach by Assaf et al.^47^. All models assume a complex constant VEM core, without considering the frequency-dependence. The parameters of material are described detailly in Ref. 47, and the panel has an in-plane dimension of 0.303 m × 0.203 m, and Fig. 5 presents the STL spectra from 250 to 4000 Hz (in 1/3 octave bands).

It is shown that the predicted STL spectrum through the present model agrees closely with Assaf et al.^47^, and has a reasonable agreement with the experiments^60^. However, it should be noted that in Assaf et al.'s model, only the core’s transverse shear deformation is considered, and neglects the fluid loading effect, which may lead to deviations in capturing the structural modal behavior compared to the present model.

## Results and discussion

This section aims to provide a thorough investigation on the problem described in section "Problem description" through the validated numerical model. Firstly, in section "Parametric effect", the parametric effect on normal incident STL is studied, and the correlation between the parameters and STL characteristics is discussed on the basis of the scanning results of free vibration analysis. Sections "Influence of boundary conditions" and "Influence of sound incident angles" explore the influence of boundary conditions and sound incident angles on STL properties. The damping contribution mechanism of constrained viscoelastic core to STL is finally analyzed in section "Mechanism of the damping contribution".

### Parametric effect

**Figure 6:**
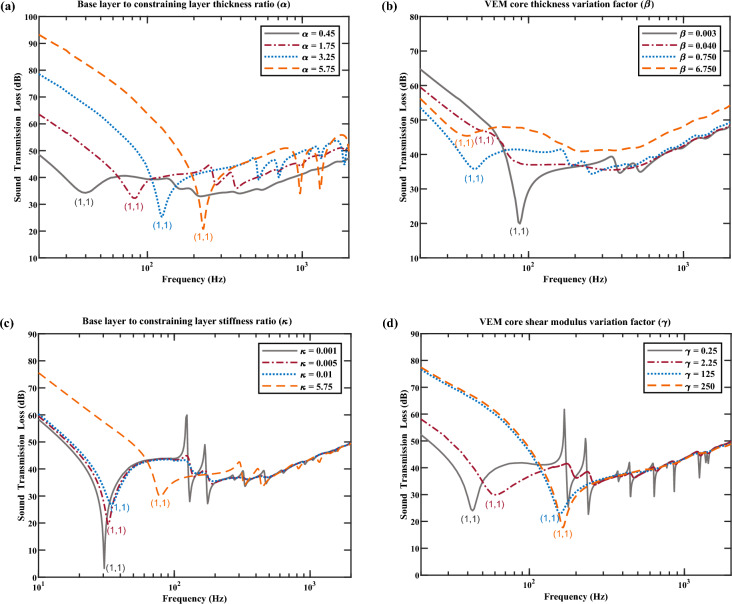
Spectra of normal incident STL with the effect of parameters variation. The parameters of symmetric CLD panel configuration are taken as the initial values, and the position of (1,1) mode in each curve is labeled. (**a**) The thickness ratio variations between base and constraining layer ($\alpha$). (**b**) The thickness variation factor of VEM core ($\beta$). (**c**) The stiffness ratio variations between base and constraining layer ($\kappa$). (**d**) The shear modulus variation factor of VEM core (${\upgamma }$).

This section focuses on investigating the STL characteristics of various CLD panel configurations to gain an overall and intuitive understanding firstly. The LW model is employed with different CLD parameter sets, such as variations in stiffness and thickness ratio of two surfaces, the viscoelastic core’s thickness and shear modulus. Figure 6 presents the corresponding results of the symmetric CLD panel configuration, and the four-edge simply supported boundary condition is adopted.

**Figure 7:**
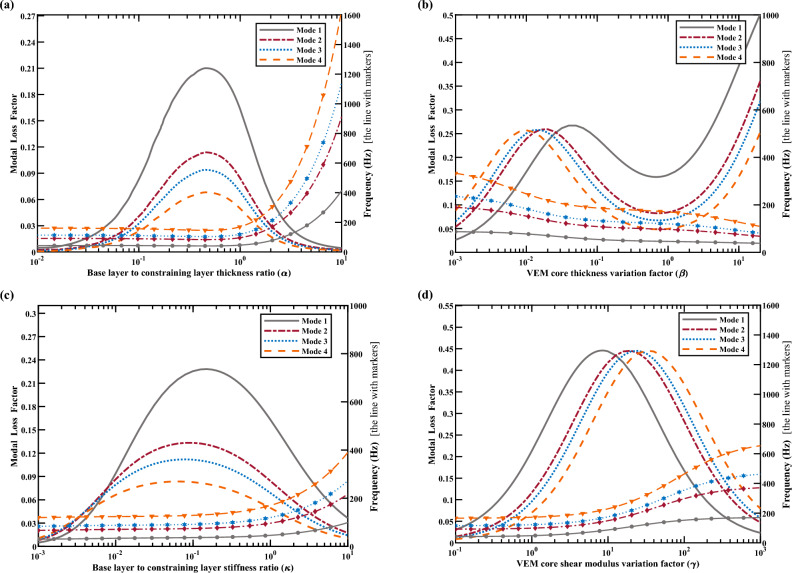
Parametric effect on the first-four order modal loss factor and natural frequency of the symmetric CLD configuration. The unmarked line represents the variation of modal loss factor. The dot-marked line represents the variation of natural frequency. (**a**) The thickness ratio variations between base and constraining layer ($\alpha$). (**b**) The thickness variation factor of VEM core ($\beta$). (**c**) The stiffness ratio variations between base and constraining layer ($\kappa$). (**d**) The shear modulus variation factor of VEM core (${\upgamma }$).

Figure 6a presents STL spectra with different base layer to constrained layer thickness ratios (represented by $\alpha$ throughout this paper) of 0.45, 1.75, 3.25, and 5.75. As $\alpha$ increases, the natural frequency of (1,1) mode and the STL in the stiffness control region lower than the (1,1) mode gradually increases. Moreover, the average performance of STL after the (1,1) mode also increases accordingly with the corresponding ratio increasing, and the dip of each STL spectra at (1,1) mode decreases. In Fig. 6b, the STL spectra with different thickness variation factors of VEM core of 0.003, 0.04, 0.75, and 6.75 are presented, and the thickness variation factor is defined as the product of the initial thickness of the VEM core and the scaling factor^47^ and is represented by $\beta$ throughout this paper. The natural frequency of (1,1) mode decreases with the increase of the thickness of VEM core, as shown in Fig. 6b. When $\beta$ = 0.003, an obvious dip of STL spectrum is observed at (1,1) mode. But when $\beta$ becomes 0.04, the STL at (1,1) mode increases distinctly so that the dip at this mode almost disappears. And the STL spectrum of $\beta =$ 0.04 is almost the same as that of $\beta =$ 0.003 at frequencies higher than 600 Hz. When the value of $\beta$ increases to be 0.75, the dip of STL spectrum at (1,1) mode becomes more apparent than that of $\beta =$ 0.04, and the STL spectrum for frequencies above 600 Hz closely resembles to the previous two spectra in Fig. 6b. When the value of $\beta$ increases to be 6.75 representing a considerably thick VEM core, the overall trend of STL spectrum significantly improves compared to those with thin VEM cores. Additionally, the dip of STL spectra at (1,1) mode only slightly decreases, and it is not very prominent. Within the stiffness control region, located at frequencies below the (1,1) mode, there is an interesting phenomenon that the STL first increases and then decreases with $\beta$ increasing from 0.003 to 0.75, but on the contrary increases with $\beta$ increasing from 0.75 to 6.75. More results will be presented in Fig. 7b to thoroughly discuss the corresponding reason. Figure 6c presents the spectra of STL with the stiffness ratio variations between base and constraining layer (represented by $\kappa$ throughout this paper) of 0.001, 0.005, 0.01, and 5.75.

It can be found that with the increase of $\kappa$, the performance of STL spectrum inside the stiffness control region lower than the (1,1) mode gradually increases, and the dips of each STL spectra at (1,1) mode increase accordingly. Within the frequencies of 100–600 Hz, and with the value of $\kappa$ increases from 0.001 to 0.01, the dips of each STL spectra gradually become less noticeable. However, when the value of $\kappa$ is further increased from 0.01 to 5.75, the dips of STL spectra become more pronounced again. When the frequency is greater than 600 Hz, the STL spectra for different values of $\kappa$ almost coincide. Finally, Fig. 6d presents the spectra of STL with different stiffness variation factors of VEM core (represented by ${\upgamma }$ throughout this paper) of 0.25, 2.25, 125, and 250. The initial value of the shear modulus of VEM is taken as 2.67e6 Pa. With the increase of ${\upgamma }$, the performance of STL spectrum inside the stiffness control region lower than the (1,1) mode gradually increases. However, the dips of STL spectrum at (1,1) mode increase with the values of ${\upgamma }$ increased from 0.25 to 2.25, and then decrease with the value of ${\upgamma }$ increase from 125 to 250, accordingly. Furthermore, there is little disparity in the STL spectrum for the value of ${\upgamma }$ equal to 125 and 250, indicating that increasing the value of ${\upgamma }$ beyond a certain point has a negligible effect on the STL performance where the VEM core’s shear modulus is comparatively large concerning its initial value. For frequencies above the natural frequency of (1,1) mode, it can be seen that the dips of STL spectrum become less pronounced with the increase of ${\upgamma }$. It should be noted that in Fig. 6a, b, the stiffness and overall weights of the CLD panels change simultaneously with the variation of $\alpha$ and $\beta$, whereas in Fig. 6c, d only the stiffness changes with the variation of $\kappa$ and ${\upgamma }$. Consequently, for higher frequency ranges dominated with the mass law (> 600 Hz), the overall performances of these four STL spectra are nearly identical in Fig. 6c or d, accordingly. The finding from Fig. 6 indicates that the structural dynamic characteristics (e.g., stiffness and modal loss factor) of finite CLD panels are altered by the parameter variations, which in turn affect the STL performance. Further insights can be obtained by studying the scanning data of the structural modal loss factor and natural frequency in Fig. 7.

Figure 7a presents the influence of $\alpha$ varying from 0.01 to 10 on the first-four order modal loss factor and natural frequency of the symmetric CLD configuration. An inverse parabolic trend is observed on the change of modal loss factor. Meanwhile, the natural frequency exhibits a slow initial increase trend followed by a sharp increment trend when $\alpha$ exceeds 1, which indicates a significant improvement in structural stiffness. This observation can be used to explain the phenomenon shown in Fig. 6a. Taking the STL performance at the natural frequency of (1,1) mode as an example, it can be found that although the natural frequency is the lowest for $\alpha =$ 0.45, the highest modal loss factor at this value results in the least significant dip of STL spectra among different values of $\alpha$. Figure 7b presents the influence of $\beta$ varying from 0.001 to 20 on the modal loss factor and natural frequency of the symmetric CLD configuration. The natural frequency decreases monotonically and slowly. However, the modal loss factor shows an interesting change. When $\beta$ is greater than 1, the large composition ratio of VEM in whole structure caused a sharp increase of modal loss factor. When $\beta$ increases from 0.001 to 1, the optimal values can be found for different modal orders to achieve relatively high modal loss factors, which is ideal for reducing the use of VEM and achieving lightweight. Within this range, the main source of structural damping is from the deformation of the VEM core. The condition of $\beta =$ 0.04 in Fig. 6b falls into this category, where a relatively large modal loss factor observed at this value results that the STL dip is almost eliminated at (1,1) mode. When the VEM core is moderately thin, the cohesive force generated by the molecular structure of the VEM is relatively weak, and the constrained surface layer on both sides is more likely to generate a large degree of deformation^61^. The structural modal loss factor is related not only to VEM proportion in total weight but also to the VEM core’s deformations. The proportion of VEM in the overall structure dominates the change of modal loss factors when the VEM is either too thin or too thick, rather than just the deformations. The curve in Fig. 7c exhibits similar trends with the influence of $\kappa$ varying from 0.001 to 10. Finally, the influence of ${\upgamma }$ varying from 0.1 to 1000 on the modal loss factor and natural frequency of the symmetric CLD panel is presented in Fig. 7d. The increase trend in natural frequency is found as ${\upgamma }$ increasing. Although the mode loss factors exhibit an anti-parabolic tendency, each mode has a different optimal value of ${\upgamma }$ to achieve the optimal modal loss factor.

Similarly, the results of parametric effect on the normal incident STL of the asymmetric CLD panel configuration, as well as the first-four order modal loss factor and corresponding natural frequency are presented in Figs. B1 and B2 in Supplementary Appendix B detailly.

To summarize, the dips in each STL spectrum in Fig. 6 and Fig. B1 are positively correlated with the corresponding value of modal loss factors in Fig. 7 and Fig. B2, and the value of modal loss factor is influenced by corresponding structural parameters investigated in this section. The impact of modal resonances on the STL of finite CLD panels can be assessed by modal loss factors. It is found that the dips of STL spectra can be mitigated with higher modal loss factors at resonant frequencies.

### Influence of boundary conditions

**Figure 8:**
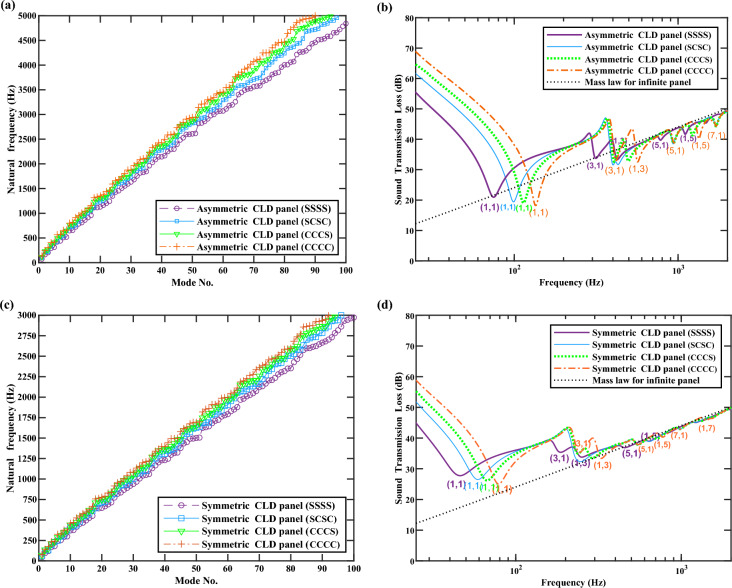
Influence of boundary condition on natural frequency and normal incident STL. Letter C and S correspond to clamped and simply supported edge, respectively, and letter combinations (SSSS, SCSC, CCCS, and CCCC) indicate the corresponding constraints applied to the four edges. (**a**) Natural frequency variation under different boundary conditions of the asymmetric CLD panel. (**b**) Normal incident STL spectra of the asymmetric CLD panel under the four constraint boundary conditions, and mass law for an equivalent weight infinite panel, respectively. (**c**) Natural frequency variation under different boundary conditions of the symmetric CLD panel. (**d**) Normal incident STL spectra of the symmetric CLD panel under the four kinds boundary conditions, and mass law for an equivalent weight infinite panel, respectively.

The influence of boundary condition on natural frequency and normal incident STL characteristics of the finite asymmetric and symmetric CLD panel is investigated here, as it is well-known that the boundary condition is one of the key factors affecting the dynamic behaviors of finite panels. The letters S or C in Fig. 8 denotes simply supported or clamped condition for one of the finite CLD panel edges, and letter combinations such as SSSS and CCCC indicate the corresponding constrained condition for all four edges. It can be found that from Fig. 8a, c, the SSSS condition exhibits the lowest constraint, which is represented by the lowest growth rate of natural frequencies, while the CCCC condition is the highest constraint, followed by the SCSC condition and the CCCS condition.

Figure 8b, d present the normal incident STL spectra of the finite asymmetric and symmetric CLD panel under four constraint boundary conditions, along with the mass law for an equivalent weight infinite panel. The STL characteristics of finite CLD panel in low frequencies (< 500 Hz) are significantly different from the mass law of infinite panel. Within the stiffness control region lower than the natural frequency of (1,1) mode, the higher structural stiffness of the CCCC condition leads to significantly higher STL compared with the SSSS condition. The STL of finite CLD panel significantly deviates from the mass law until frequencies reach the natural frequency of (1,3) mode, as the result of the drastic modal resonances. In middle frequency range (500–2000 Hz), The SSSS condition demonstrates a sound insulation performance close to mass law, where the effect of modal resonance is relatively inapparent. The overall sound insulation performance of the symmetric panel is superior to that of the asymmetric panel. Meanwhile, the significant dips of STL spectrum caused by higher-order modes, such as (5,1), (1,5), and (7,1) modes, are also observed for the CCCC condition for the asymmetric CLD panel. The structural resonance modes can have an adverse impact on STL within the low and middle frequencies, which is manifested as a series of dips in the STL spectrum. On one hand, the constraint strength of boundary conditions impacts the structural apparent bending stiffness, leading to variations in the natural frequencies of structural resonance modes. Consequently, the natural frequency of (1,1) mode is the highest under the boundary condition of CCCC, which contributes to the highest sound insulation performance of the structures in the stiffness-controlled region. On the other hand, the constraint strength of boundary conditions also influences the modal loss factor of the same CLD panel configuration. A comparison between Fig. 8b or d reveals that, for both asymmetric or symmetric CLD panels, stronger boundary constraints lead to a more pronounced reduction of the dips in STL spectrum at resonance frequencies, particularly at the natural frequency of (1,1) mode. This phenomenon primarily stems from the constraints imposed by boundary conditions on the relative interlayer’s deformations of the CLD panel, thereby influencing the deformations of VEM core, which is reflected in the value of the structural modal loss factors. Smaller modal loss factors result in a relatively lower contribution to enhancing the dips of STL spectrum at resonance frequencies.

In short, it is found that the primary distinction among the four boundary conditions is the different degrees of constraint strength, which affects the natural frequencies of structural resonant modes and the magnitude of modal loss factors. The influence mechanism on STL of boundary conditions remains consistent. To highlight the primary focus of this study, sections "Influence of sound incident angles" and "Mechanism of the damping contribution" exclusively concentrate on the lowest and highest constraint boundary conditions (SSSS and CCCC), conducting exhaustive investigations into the influence and contribution mechanism of constrained VEM core on STL. The findings drawn under these two boundary conditions are of general applicability.

### Influence of sound incident angles

The STL characteristics of three panel configurations proposed in section "Problem description" are investigated with varying the sound incident angles. The elevation angle ($\theta$) is varied as $0^{ \circ }$, $45^{ \circ }$, and $78^{ \circ }$, while the azimuth angle ($\phi$) is fixed as $0^{ \circ }$.

**Figure 9:**
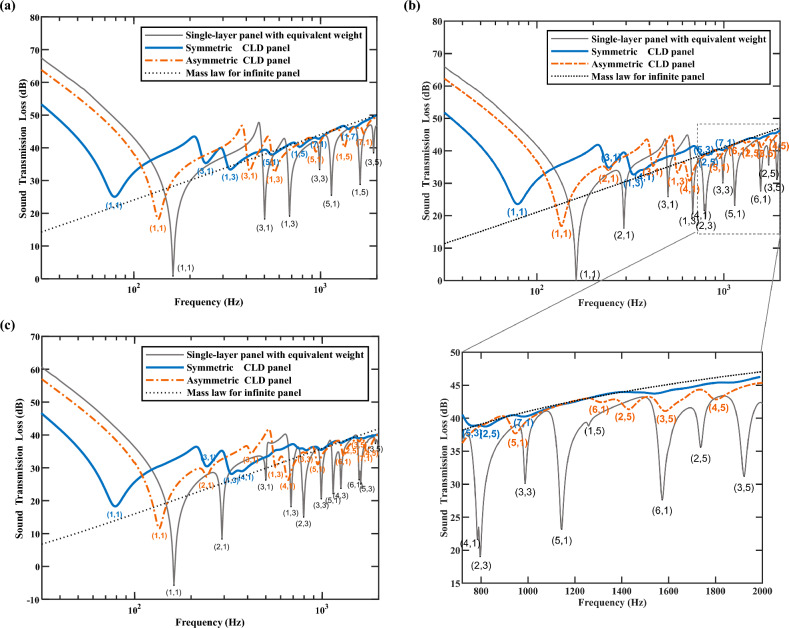
Influence of sound incident angles on STL of the three panel configurations under four edges clamped condition. (**a**) Spectra of normal incident STL ($\theta = 0^{ \circ } , \, \phi = 0^{ \circ }$). (**b**) Spectra of oblique incident STL ($\theta = 45^{ \circ } , \, \phi = 0^{ \circ }$) with a local detail view focus on the modal coupling effect at mid-frequency bands. (**c**) Spectra of oblique incidence incident STL ($\theta = 78^{ \circ } , \, \phi = 0^{ \circ }$).

Figure 9 presents the STL spectra of three panel configurations under four edges clamped condition in narrow bands, and the mass law for an equivalent weight infinite panel, respectively. The orders of structural modes resonances that significantly affect the STL performance are labeled. The structural loss factor of the single-layer steel panel remains constant at a relatively small value ($\eta =$ 0.001), and thus both low-order modes and high-order modes lead to evident dips of STL spectra.

In this case, the modal loss factors for the first-three odd-odd order modes, namely (1,1), (3,1), and (1,3) modes, of the symmetric CLD panel are 0.069, 0.030, and 0.023, respectively, and those for the asymmetric CLD panel are 0.024, 0.011, and 0.008, respectively. The asymmetric CLD panel’s stiffness is higher, resulting in smaller modal loss factors and higher STL in the stiffness control region. The first-three odd-odd modes have a strong adverse effect on the STL of both CLD configurations, no matter the situation of normal and oblique incidence. A larger modal loss factor can enhance, or even eliminate, the dip of STL spectra caused by dramatic resonance transmission components. Normal plane sound wave incidence results in even sound pressure distribution on the structural surface layer, exciting only odd-odd modes (Fig. 9a). As the frequencies exceed the natural frequencies of (1,3) modes, the overall STL performances of the two CLD configurations are much closer to that of the mass law for an equivalent weight infinite panel. Meanwhile, as is shown in Fig. 9b, c, even–odd modes, such as (2,1) and (4,1) modes, can also cause significant reductions in STL of the three panel configurations under oblique incident conditions. And the adverse impact of some higher-order modes on the STL of the three panel configurations, such as (5,1) and (3,5) modes, cannot be disregarded. It should be noted that in mid-frequency bands, a high density of structural modes exists, which can result in inter-modal coupling that causes a significant decrease in STL over a relatively broad frequency range^62^. The local detail view of the STL spectra from 750 to 2000 Hz in Fig. 9b clearly shows this phenomenon. In frequencies where the (4,1) and (2,3) modes or the (6,1), (2,5), and (3,5) modes are inter-modally coupled, the STL of single-layer steel panel is significantly lower than that predicted by the mass law. Meanwhile, the STL dips for both CLD configurations are not as severe as those for the single-layer panel due to their higher modal loss factors. The amplitude of lower-order mode is generally higher than that of higher-order mode. Thus, for higher frequencies, a smoother narrow-band STL spectra can be achieved with relatively small modal loss factors, which can closely approximate or even achieve the ideal prediction according to the mass law of infinite panel. Since the spectra of most sound sources are unique and continuous in the real world, the CLD treatment is of great practical importance. For example, CLD panels have the potential to effectively isolate the low-frequency line-spectrum radiation noise of power transformers.

### Mechanism of the damping contribution

**Figure 10:**
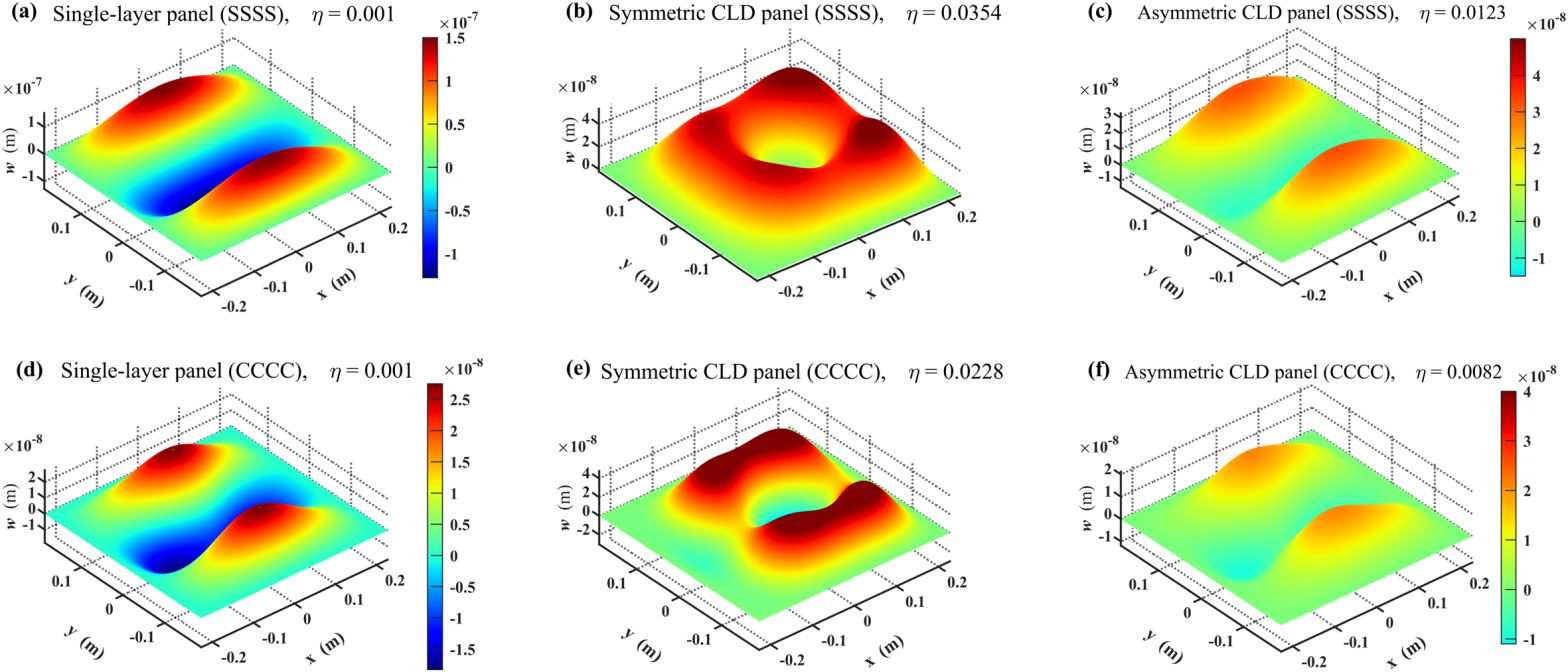
Deflected bending modal shapes of the three panel configurations under oblique plane sound excitation ($\theta { = 78}^{ \circ } , \, \phi { = 0}^{ \circ }$) controlled by the (1,3) mode. Colormaps are normalized to the same range for (**a,b**), as well as (**e,f**), respectively. (**a–c**) The three panel configurations under four edges simply supported condition (SSSS). (**d–f**) The three panel configurations under four edges clamped condition (CCCC).

In this section, the deflected bending modal shapes of the three panel configurations, controlled by the (1,3) mode, are used to provide a clearer explanation of the mechanism of damping contribution to the STL performance. As is shown in Fig. 10, the positive normal displacement direction ($w > 0$) indicates sound incident direction. Overall, the placement of VEM core significantly reduces the normal displacement amplitude of the two CLD panels by about an order of magnitude. The form of deflected modal shape is almost the same as that in free vibration for the single-layer steel panel, whereas those for the two CLD configurations become complex with nonregular node lines. The deflected bending modal shapes of the symmetric CLD panel at (1,3) mode reveal a greater normal displacement amplitude distribution towards the sound incidence direction and a smaller amplitude distribution towards the sound transmission direction. As a result, a larger proportion of sound power is reflected and correspondingly, a smaller proportion of sound power is transmitted. This leads to the increase of STL as deduced from Eq. (9). Despite the fact that the deflected bending modal shapes of the asymmetric CLD panel change only slightly, its modal loss factor are still higher compared to the single-layer steel panel. Thus, the dip of STL spectrum here has been improved to some extent.

In all, the mechanism by which VEM contributes to STL can be summarized in two points. Firstly, the normal displacement amplitude of the deflected bending modal shapes is attenuated. Secondly, the distribution form of normal displacement amplitudes is changed, resulting in a larger proportion of reflected sound power and a correspondingly smaller proportion of transmitted sound power. These two mechanisms lead to an improvement in sound insulation performance at modal resonances.

## Conclusions

This study explores the damping contribution of constrained viscoelastic core on airborne sound insulation performance of finite CLD panels using a coupled LW approach based on a generalized HSDT hypothesis. The present approach considers the VEM’s frequency-dependence and provides an accurate prediction of modal loss factors. Several parameters that have a significant influence on STL of CLD panels are investigated extensively. These parameters include variations in stiffness and thickness ratio of two surface layers, the VEM core’s thickness and shear modulus, as well as the sound incident angles and boundary conditions. An increase in structural stiffness results in a higher natural frequency of the (1,1) mode, which in turn leads to a higher STL in the stiffness control region. The dips of STL spectra caused by dramatic resonance transmission components can be enhanced, or even eliminated, by larger modal loss factors. Moreover, the STL characteristics of finite CLD panels under different boundary conditions are quite different from those of the mass law for an equivalent weight infinite panel, especially below the natural frequency of (1,3) mode, where the lowest constraint condition is four edges simply supported and the highest constraint condition is four edges clamped. Above the natural frequency of (1,3) mode, the smooth STL spectra close to the mass law are observed for finite CLD panels. The modal resonances cause significant dips of STL spectra for finite single-layer steel panel in all frequency bands at arbitrary incidence angles. The damping of VEM core can mitigate the adverse modal resonance impact on STL of finite CLD panels, as well as the broadband STL decrease due to inter-modal coupling in mid-frequencies. Results show that the damping contribution mechanisms of VEM core can be divided into two aspects: (i) the reduction of modal amplitude through the dissipation of vibration energy by various deformations of VEM core, and (ii) the change of bending modal shapes, which results in a smaller proportion of transmitted sound power. Thus, the CLD treatment is a concise and effective way to achieve stable sound insulation performance. The free vibration parametric study allows for identifying the maximum modal loss factor. Further, this can be combined with multi-objective optimization algorithms to obtain optimal configurations for high sound insulation and lightweight.

### Supplementary Information

Supplementary Information.

## Data Availability

The datasets generated during the current study are available from the corresponding author on reasonable request.
