# Supplementary material for: Damping contribution of viscoelastic core on airborne sound insulation performance of finite constrained layer damping panels at low and middle frequencies
Source: Sci Rep. 2023 Sep 20;13:15556. doi: 10.1038/s41598-023-42391-9 (PMC10511544; doi:10.1038/s41598-023-42391-9)
Supplement: Supplementary file 1 — Supplementary Information. [file 41598_2023_42391_MOESM1_ESM.pdf]

Supplementary Information *for*:

Damping contribution of viscoelastic core on airborne sound insulation performance of finite constrained layer damping panels at low and middle frequencies

Bo Wang[1], Hequn Min[1,*]

[1]*Key Laboratory of Urban and Architectural Heritage Conservation, Ministry of Education, School of Architecture, Southeast University, 2# Sipailou, Nanjing 210096, China*

*corresponding author: hqmin@seu.edu.cn

APPENDIX A

For the coupled structure-sound interaction system equation, the 3D discretized acoustic wave equation of sound field can be written as

$$\sum_e\left[\iiint_{\Omega_A^e}\frac{1}{c^2}\delta p\frac{\partial^2 p}{\partial t^2}d\Omega\right]+\sum_e\left[\iiint_{\Omega_A^e}(\nabla\cdot\delta p)(\nabla p)d\Omega\right]+\sum_e\left[\iint_{\Sigma_{SA}^e}\rho_0\delta p\mathbf{n}^T\mathbf{u}d\Sigma\right]$$
$$-\sum_e\left[\iint_{\Sigma_{AA}^e}\mathbf{n}^T\delta p(\nabla p)d\Sigma\right]=0 \tag{A.1}$$

where Ω_A^e, Σ_{SA}^e represent the element of the sound field, fluid-structure interaction boundary, Σ_{AA}^e is the element of sound field boundary distributed on both sides of the structure.

By introducing bilinear interpolation test functions, the variation of sound pressure p and structural displacement $\mathbf{u}$ can be expressed as

$$p(x,y,z)=\mathbf{N}_P^T\mathbf{P}^e,\qquad \mathbf{u}=\mathbf{N}_U\mathbf{U}^e \tag{A.2}$$

where $\mathbf{N}_P$, $\mathbf{N}_U$ are the node interpolation functions for each element vector. Similarly, the matrix used in Eq. (A.1) can be written as

$$\mathbf{M}_A = \frac{1}{c^2}\iiint\limits_{\Omega_A} \mathbf{N}_P \mathbf{N}_P^T d\Omega \ , \quad \mathbf{K}_A = \iiint\limits_{\Omega_A} \left(\nabla \mathbf{N}_P^T\right)\left(\nabla \mathbf{N}_P^T\right)^T d\Omega$$

$$\mathbf{M}_S = \rho_S \iiint\limits_{\Omega_S} \mathbf{N}_U \mathbf{N}_U^T d\Omega \ , \quad \mathbf{K}_S = \iiint\limits_{\Omega_S} \mathbf{B}\mathbf{D}\mathbf{B}^T d\Omega \tag{A.3}$$

$$\mathbf{C}_{SA} = \iint\limits_{\Sigma_{SA}} \mathbf{N}_P \mathbf{n}^T \mathbf{N}_U^T d\Sigma \ , \quad \mathbf{F}_A = \mathbf{C}_{SA}^T \mathbf{P}$$

where ρ_S is the density of the CLD plate, $\mathbf{B}$ is the coefficient matrix connecting stress and nodal displacement, $\mathbf{D}$ is the elastic matrix representing the constitutive relationship of the materials.

The dynamic equations of the CLD plate can be derived using the Hamilton's principle

$$\delta \int_{t_1}^{t_2} \left(U - T + W\right) dt = 0 \tag{A.4}$$

where U, T are the strain energy and kinetic energy, respectively. W is the virtual work done by the external forces, which can be expressed as

$$W = \mathbf{u}^T \mathbf{F}_A \tag{A.5}$$

The strain energy and kinetic energy for n elements can be written as

$$\begin{cases} U = \frac{1}{2}\sum_{p=1}^{n}\left[\mathbf{U}_p^e\right]^T \left[\mathbf{K}_b\right]_p \left[\mathbf{U}_p^e\right] + \frac{1}{2}\sum_{p=1}^{n}\left[\mathbf{U}_p^e\right]^T \left[\mathbf{K}_c\right]_p \left[\mathbf{U}_p^e\right] \\ \quad + \frac{1}{2}\sum_{p=1}^{n}\left[\mathbf{U}_p^e\right]^T \left[\mathbf{K}_t\right]_p \left[\mathbf{U}_p^e\right] \\ T = \frac{1}{2}\sum_{p=1}^{n}\left[\dot{\mathbf{U}}_p^e\right]^T \left[\mathbf{M}_b\right]_p \left[\dot{\mathbf{U}}_p^e\right] + \frac{1}{2}\sum_{p=1}^{n}\left[\dot{\mathbf{U}}_p^e\right]^T \left[\mathbf{M}_c\right]_p \left[\dot{\mathbf{U}}_p^e\right] \\ \quad + \frac{1}{2}\sum_{p=1}^{n}\left[\dot{\mathbf{U}}_p^e\right]^T \left[\mathbf{M}_t\right]_p \left[\dot{\mathbf{U}}_p^e\right] \end{cases} \tag{A.6}$$

where $\mathbf{K}_t$, $\mathbf{K}_b$, and $\mathbf{K}_c$ are the elemental stiffness matrix of the top, bottom and the core, respectively. $\mathbf{M}_t$, $\mathbf{M}_b$, and $\mathbf{M}_c$ are the elemental mass matrix top, bottom and the core, respectively. Then, the total structural stiffness and mass matrix can be given by

$$\mathbf{K}_S = \mathbf{K}_b + \mathbf{K}_c + \mathbf{K}_t \tag{A.7}$$

$$\mathbf{M}_S = \mathbf{M}_b + \mathbf{M}_c + \mathbf{M}_t \tag{A.8}$$

Finally, the dynamic equation can be derived through connecting all elemental mass and stiffness matrices to global coordinate system and is given by

$$\mathbf{M}_S\ddot{\mathbf{u}} + \mathbf{K}_S\mathbf{u} = \mathbf{F}_A \tag{A.9}$$

where $\mathbf{M}_S$, $\mathbf{K}_S$, and $\mathbf{F}_A$ are the same variables defined in Eq. (A.1). $\mathbf{u}$ and $\ddot{\mathbf{u}}$ are the structural displacement vector and the second-order time derivative of displacement, respectively.

APPENDIX B

The results of parametric effect on the normal incident STL of the asymmetric CLD panel configuration, as well as the first-four order modal loss factor and corresponding natural frequency are presented in Fig. B1 and Fig. B2 in Appendix B detailly.

Figure B1(a) presents STL spectra with different base layer to constrained layer thickness ratios (represented by α throughout this paper) of 0.3, 2.2, 73, and 550. Only variations in the corresponding thickness of the constrained layer are made, thus a decrease in the ratio (α) implies an increase in the thickness of the constrained layer. As the value of α increases from 0.3 to 2.2 and from 2.2 to 550, the dips of each STL spectra at (1,1) mode first increase and then descend accordingly. Moreover, the average performance of STL after the (1,1) mode also decreases accordingly with the corresponding ratio increasing. In Fig. B1(b), the STL spectra with different thickness variation factors of VEM core of 0.003, 0.04, 0.75, and 6.75 are presented, and the thickness variation factor is defined as the product of the initial thickness of the VEM core and the scaling factor and is represented by β throughout this paper. The natural frequency of (1,1) mode decreases with the increase of the thickness of VEM core, as shown in Fig. B1(b). When β= 0.003, an obvious dip of STL spectrum is observed at (1,1) mode. And the STL spectrum of β= 0.04 is almost the same as that of β= 0.003 at frequencies higher than 600 Hz. When the value of β increases to be 0.75, the dip of STL spectrum at (1,1) mode becomes less apparent than that of β= 0.04, and the STL spectrum for frequencies above 600 Hz closely resembles to the previous two spectra in Fig. B1(b). When the value of β increases to be 6.75 representing a considerably thick VEM core, the overall trend of STL spectrum significantly improves compared to those with thin VEM cores. Additionally, the dip of STL spectra at (1,1) mode only slightly decreases, and it is almost eliminated. Within the stiffness control region, located at frequencies below the (1,1) mode, there is an interesting phenomenon that the STL first increases and then decreases with β increasing from 0.003 to 0.75, but on the contrary increases

with β increasing from 0.75 to 6.75. More results will be presented in Fig. B2(b) to thoroughly discuss the corresponding reason.

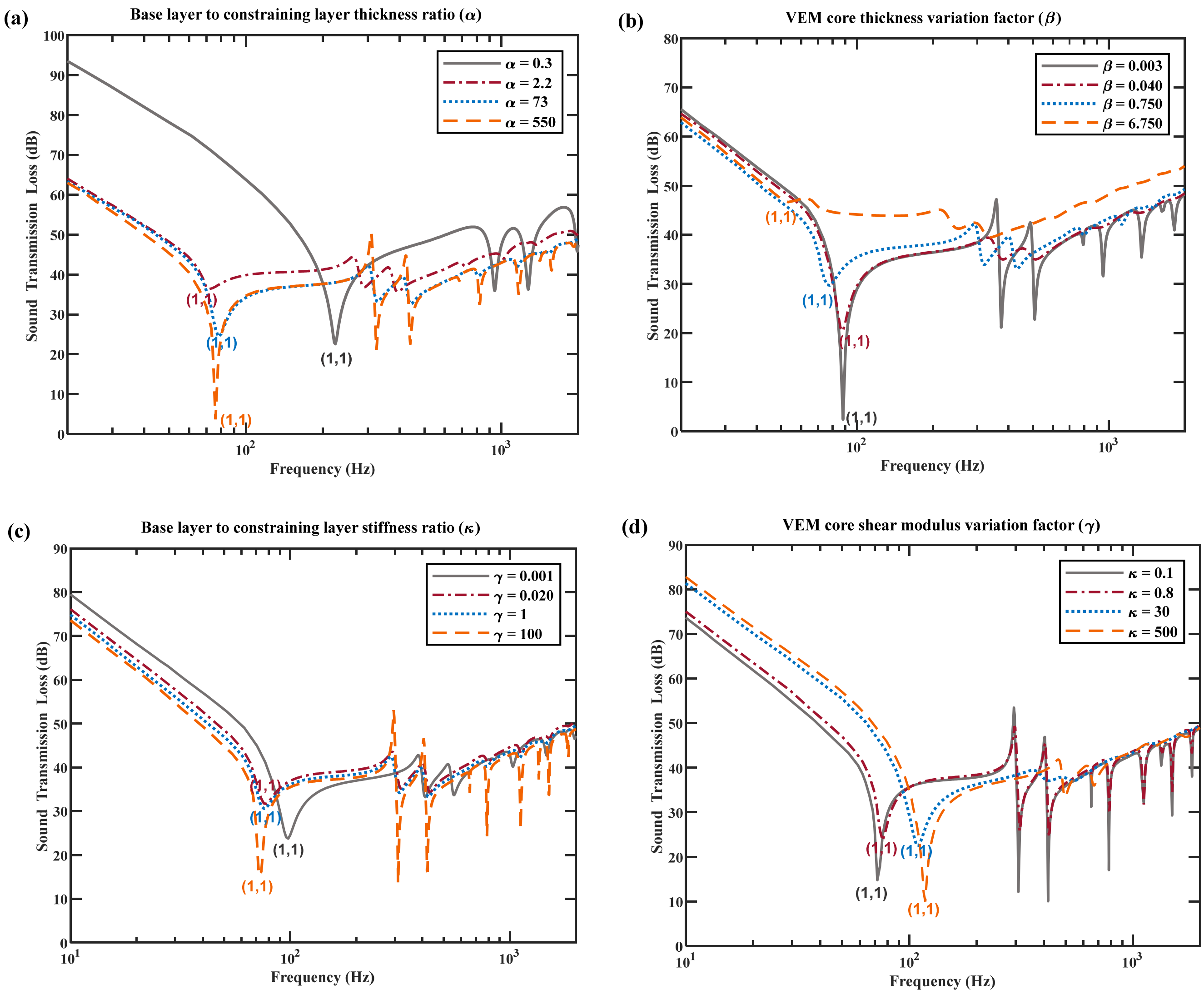

Figure B1. Spectra of normal incident STL with the effect of parameters variation. The parameters of asymmetric CLD panel configuration are taken as the initial values, and the position of (1,1) mode in each curve is labeled. (a) The thickness ratio variations between base and constraining layer (α). (b) The thickness variation factor of VEM core (β). (c) The stiffness ratio variations between base and constraining layer (κ). (d) The shear modulus variation factor of VEM core (γ).

Figure B1(c) presents the spectra of STL with the stiffness ratio variations between base and constraining layer (represented by κ throughout this paper) of 0.001, 0.02, 1, and 100. It can be found that as the value of κ increases from 0.001 to 0.02 and from 0.02 to 100, the dips of each STL spectra at (1,1) mode first increase and then descend accordingly. Meanwhile, with the increase of κ, the performance of STL spectrum inside the stiffness control region lower than the (1,1) mode gradually decreases. Within the frequencies of 100 - 600 Hz, and with the value of κ increases from 0.001 to 0.02, the dips of each STL spectra gradually become less noticeable. However, when the value of κ is further increased from 0.02 to

100, the dips of STL spectra become more pronounced again. When the frequency is greater than 600 Hz, the overall trendline of each STL spectra for different values of κ are almost coincide. Finally, Fig. B1(d) presents the spectra of STL with different stiffness variation factors of VEM core (represented by γ throughout this paper) of 0.1, 0.8, 30, and 500. The initial value of the shear modulus of VEM is taken as 2.67e6 Pa. With the increase of γ, the performance of STL spectrum inside the stiffness control region lower than the (1,1) mode gradually increases. However, the dips of STL spectrum at (1,1) mode increase with the values of γ increased from 0.1 to 0.8, and then decrease with the value of γ increased from 30 to 500, accordingly. Furthermore, there is little disparity in the STL spectrum for the value of γ equal to 30 and 500, indicating that increasing the value of γ beyond a certain point has negligible effect on the STL performance where the VEM core's shear modulus is comparatively large concerning its initial value.

It should be noted that in Fig. B1(a) and B1(b), the stiffness and overall weights of the CLD panels change simultaneously with the variation of α and β, whereas in Fig. B1(c) and B1(d) only the stiffness changes with the variation of κ and γ. Consequently, for higher frequency ranges dominated mainly by the mass law (> 600 Hz), the overall performances of these four STL spectra are nearly identical in Fig. B1(c) and B1(d), accordingly. The finding from Fig. B1 indicates that the structural dynamic characteristics (e.g., stiffness and modal loss factor) of finite CLD panels are altered by the structural parameter variations, which in turn affect the STL performance. Further insights can be obtained by studying the scanning data of the structural modal loss factor and natural frequency in Fig. B2.

Figure B2(a) presents the influence of α varying from 0.01 to 1000 on the first-four order modal loss factor and natural frequency of the asymmetric CLD panel configuration. An approximate inverse parabolic trend is observed on the change of modal loss factor. Meanwhile, the natural frequency exhibits a sharp increment trend followed by a slow initial increase trend when α exceeds 1, which indicates a significant decreasing in structural stiffness. This observation can be used to explain the phenomenon shown in Fig. B1(a). Taking the STL performance at the natural frequency of (1,1) mode as an example, it can be found that although the natural frequency is the lowest for $\alpha =$ 0.3, the highest modal loss factor at this value results in the least significant dip of STL spectra among different values of α. Fig. B2(b) presents the influence of β varying from 0.001 to 10 on the modal loss factor and natural frequency of the asymmetric CLD configuration. The natural frequency decreases monotonically and slowly. However, the modal loss factor shows an interesting change. When β is greater than 1, the large composition ratio of VEM in whole structure caused a sharply increase of modal loss factor. When β increases from 0.001 to 1, the optimal values can be found for different modal orders to achieve relatively high modal loss factors, which is ideal for reducing the use of VEM and achieving lightweight. Within this range, the main source of

structural damping is from the deformation of the VEM core. The condition of $\beta =$ 0.04 in Fig. B1(b) falls into this category, where a relatively large modal loss factor observed at this value results that the STL dip is almost eliminated at (1,1) mode. When the VEM core is moderately thin, the cohesive force generated by the molecular structure of the VEM is relatively weak, and the constrained surface layer on both sides is more likely to generate a large degree of deformation. The structural modal loss factor is related not only to VEM proportion in total weight but also to the VEM core's deformations. The proportion of VEM in the overall structure dominates the change of modal loss factors when the VEM is either too thin or too thick, rather than just the deformations.

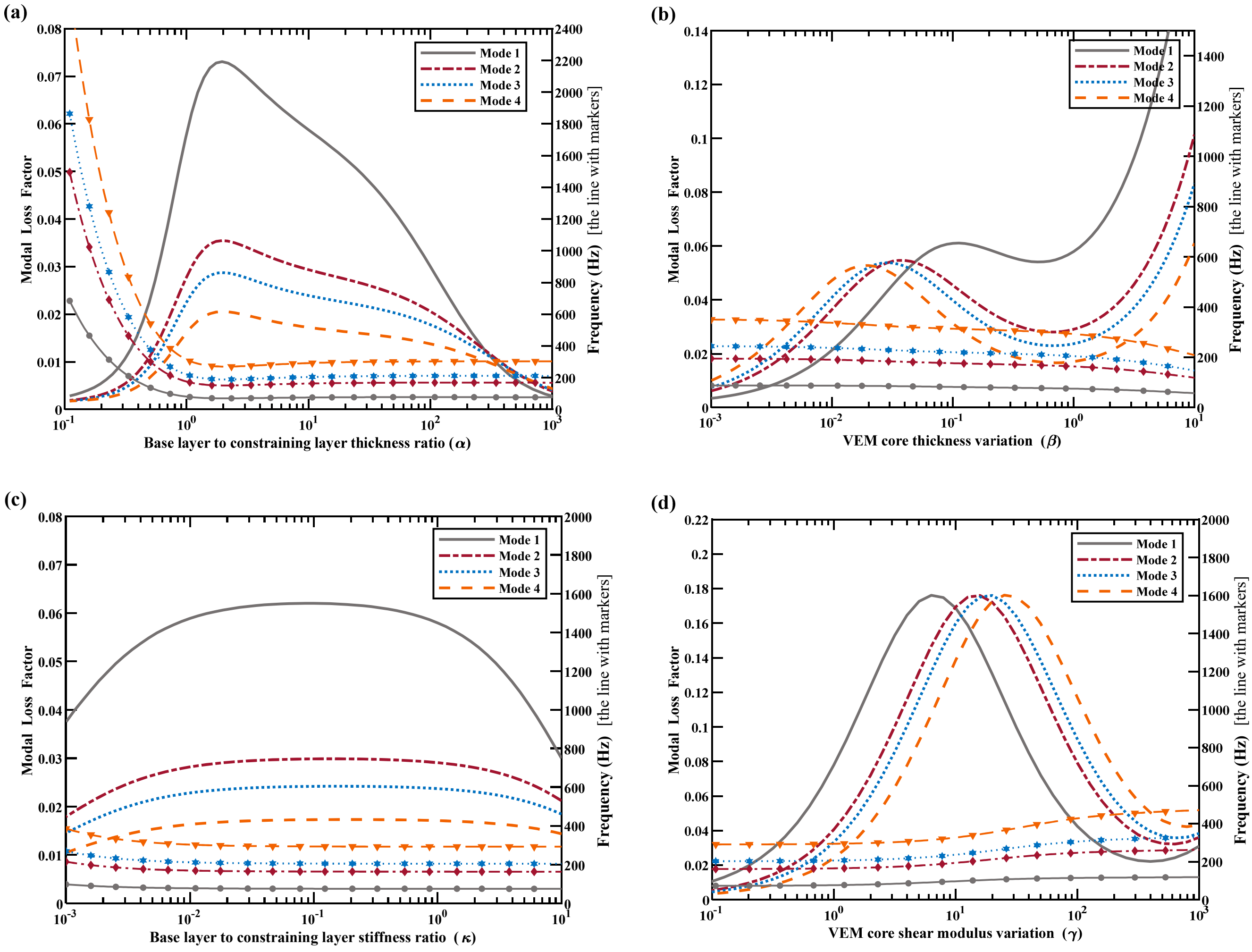

Figure B2. Parametric effect on the first-four order modal loss factor and natural frequency of the asymmetric CLD panel configuration. The unmarked line represents the variation of modal loss factor. The dot-marked line represents the variation of natural frequency. (a) The thickness ratio variations between base and constraining layer (α). (b) The thickness variation factor of VEM core (β). (c) The stiffness ratio variations between base and constraining layer (κ). (d) The shear modulus variation factor of VEM core (γ).

The curve in Fig. B2(c) exhibits similar trends with the influence of κ varying from 0.001 to 10, where the variation of the modal loss factors and natural frequency are less sensitive to the value of κ,

comparing with the symmetric CLD panel configuration. Finally, the influence of γ varying from 0.1 to 1000 on the modal loss factor and natural frequency of the asymmetric CLD panel is presented in Fig. B2(d). The increase trend in natural frequency is found as γ increasing. Although the mode loss factors exhibit an anti-parabolic tendency, each mode has a different optimal value of γ to achieve the optimal modal loss factor.
